# Fish Oil Oleogels with Wax and Fatty Acid Gelators: Effects on Microstructure, Thermal Behaviour, Viscosity, and Oxidative Stability

**DOI:** 10.3390/gels11090723

**Published:** 2025-09-10

**Authors:** Le Thuy Truong, Wilhelm Robert Glomm, Peter Patrick Molesworth

**Affiliations:** Department of Biotechnology and Nanomedicine, SINTEF Industry, 7491 Trondheim, Norway; wilhelm.glomm@sintef.no (W.R.G.); peter.molesworth@sintef.no (P.P.M.)

**Keywords:** oleogel, salmon oil, cod liver oil, microencapsulation, oxidative stability, in vitro digestion

## Abstract

Encapsulation of fish oil within oleogels can potentially prevent oxidation and enable its use in food with programmable release within the gastrointestinal tract. Here, we report on the formation of oleogels from two different fish oils—salmon oil (SO) and cod liver oil (CLO)—using different concentrations of either rice bran wax (RBW) or myristic acid (MA) as gelators. The gels were assessed with respect to their structural, thermal, viscosity, digestive, and oxidative properties. Polarized light microscopy (POM) revealed that RBW consistently produced dense, interconnected crystalline networks across both oils, while MA formed larger, spherulitic crystals that were more sensitive to the oil type. This was further supported by time-lapse imaging, showing faster crystal growth of MA in cod liver oil. Viscosity studies indicate that the molecular weight and concentration of gelator, as well as the type of fish oil (SO vs. CLO), significantly impact the shear stability of the oleogels. Thermal and viscosity analyses confirmed that RBW-based oleogels exhibited higher crystallization temperatures and stronger viscoelastic behaviour. Based on oxidative stability measurements—as measured by peroxide value (PV) analysis—encapsulation within oleogels does not lead to significant oxidation of the fish oils and also attenuates further oxidation upon storage. The fish oil oleogels were stable when exposed to either simulated gastric or intestinal fluids (SGF and SIF, respectively), but decomposed after sequential exposure first to SGF and then to SIF. These findings could broaden the range of food products which can be fortified with fish oils.

## 1. Introduction

Fish oil is a healthy fat source with high nutritional value, which is known to have a number of well-documented health benefits, including a decrease in blood fat content, reducing inflammation, preventing cardiovascular diseases, and mitigating neurodevelopmental defects [1]. However, fish oil is prone to oxidation by reactive oxygen species, which leads to off-flavours. Consequently, fish oil is not widely used as an additive in food products [2]. The oxidative stability of fish oils can be enhanced either by the use of an antioxidant or by encapsulation within a delivery system such as an emulsion, microcapsules, or oleogels [3]. Encapsulation has been shown to prevent or attenuate oxidation of fish oil, and physically entrap odorous oxidation products [4].

Oleogels (also known as organogels) are oil-continuous analogues of hydrogels, i.e., the oil forms a continuous phase within a three-dimensional network formed by an oleogelator [5]. A number of different gelators have been reported for oleogels, including low-molecular-weight gelators such as stearic acid and high-molecular-weight gelators such as plant waxes [6]. Rice bran wax (RBW), sunflower wax, candelilla wax, and beeswax have been identified as having a number of advantages, including availability, low cost, and gelling ability [7]. Structuring of edible oils with an oleogelator to replace harmful trans and saturated fats has become a growing research area [8]. Oleogels have also been reported to attenuate oxidation of fish oil, although contradictory reports exist on the oxidation rate of oleogels compared to bulk oil [9,10]. While fish oil oleogels are considered to be promising alternatives to saturated fats in, e.g., margarine, spreads, and shortenings [9,11,12], they have also been reported to oxidize faster than the parent food product, e.g., in the case of margarine [9].

In order to implement fish oil oleogel technology into a variety of food products, understanding how the gelator and processing conditions affect the oxidation of the oil is of critical importance. Moreover, the oleogel should remain stable in the food product and during the first steps of the digestion process, i.e., while masking the fish oil from the taste receptor cells; otherwise, the technology will fail to mask any unwanted flavour and odour from the consumer. If the oleogel technology can protect fish oil from oxidation, it would provide a means to overcome many of the challenges associated with fish oil fortification. Thus, in addition to preventing oxidation of the oil, more work must be conducted to elucidate the stability and degradation of fish oil oleogels during digestion. Once elucidated, this could also allow for the design of oleogels with programmable release in the gastrointestinal tract, i.e, in the small intestine.

Despite the growing interest in oleogels, knowledge gaps remain regarding how different gelators (high vs. low molecular weight) and marine oils influence gel formation, microstructure, and functional stability. In particular, few studies have systematically compared wax- and fatty acid-based oleogels in terms of their microstructural, thermal, viscosity, digestive, and oxidative properties.

The objective of this study was to systematically evaluate the structuring performance of rice bran wax (RBW) and myristic acid (MA) in two marine oils: salmon oil (SO) and cod liver oil (CLO). We focused on characterizing their microstructures using polarized optical microscopy (POM), thermal behaviour using differential scanning calorimetry (DSC), viscosity properties using shear sweeps, stability under simulated gastric and intestinal digestion, and long-term oxidative stability via peroxide value measurements.

Rice bran wax-based oleogels formed denser, more cohesive crystal networks, with higher crystallization temperatures, higher viscosity, and superior structural stability compared to MA-based systems, which exhibited larger but less interconnected spherulites. Time-resolved microscopy showed faster and more extensive crystallization of MA in CLO than in SO, highlighting its sensitivity to oil composition. In contrast, RBW maintained consistent morphology across both oils, confirming its robustness. Both gelators significantly improved oxidative stability over 31 weeks, with no major differences in PV. In vitro digestion further revealed that RBW gels offered better resistance to structural breakdown. These findings support the use of both gelators—particularly RBW—for designing oleogels tailored to deliver functional lipids.

## 2. Results and Discussion

### 2.1. Microstructure of Oleogels

**Figure 1:**
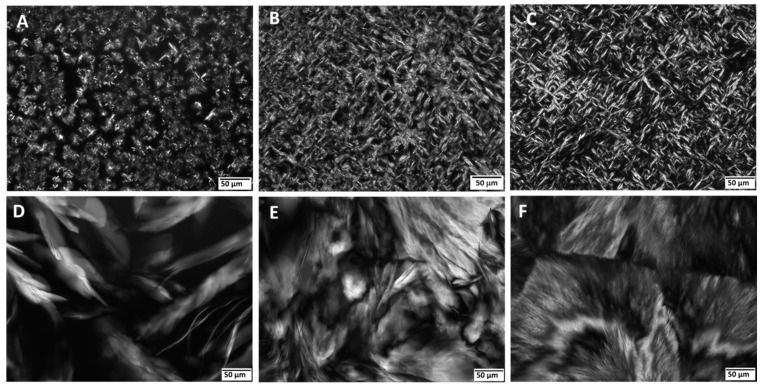
Microstructure of oleogels with fish oil under a polarized optical microscopy (scale bar 50 µm): SO 5% RBW (**A**), SO 10% RBW (**B**), SO 15% RBW (**C**), SO 5% MA (**D**), SO 10% MA (**E**), and SO 15% MA (**F**).

The microstructure of the oleogels observed by polarized optical microscopy (POM) shows differences in the crystallization patterns and network formation between RBW and myristic acid as gelators in salmon oil. Rice bran wax-based oleogels (Figure 1A–C) show a clear concentration-dependent evolution in crystal morphology. At 5% RBW (Figure 1A), the microstructure consists of sparsely distributed, small crystals. Increasing the RBW concentration to 10% (Figure 1B) and 15% (Figure 1C) results in a denser network of elongated, needle-like crystals forming a more continuous and interconnected three-dimensional structure. This structural densification is indicative of stronger intermolecular interactions and a higher degree of crystallinity, which contribute to superior mechanical strength and thermal stability. In contrast, MA-based oleogels (Figure 1D–F) show significantly different morphologies. At 5% MA (Figure 1D), the oleogel is composed of larger, flake-like or spherulitic crystals, loosely distributed throughout the oil matrix. At higher concentrations (10% and 15% MA; Figure 1E,F), the crystal size increases, and more angular, irregular shapes appear. The MA-based oleogel networks remain discontinuous and less cohesive than those formed with RBW. The visual evidence from POM suggests that RBW forms a denser and more interconnected crystalline scaffold, which is generally associated with higher oil-binding capacity and mechanical strength in oleogels [13,14]. In contrast, MA tends to form larger, more isolated crystal domains that result in a weaker, discontinuous network with less efficient oil entrapment, as similarly observed in fatty acid-based oleogels [14]. These microstructural differences are known to influence functional properties such as spreadability, melting behaviour, and oxidative stability in food systems.

**Figure 2:**
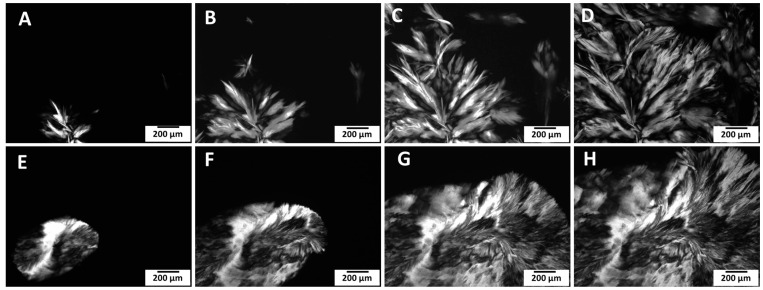
Development of crystal structures in myristic acid-based oleogels (10% MA) prepared with different fish oils, observed under polarized light microscopy over time (scale bar: 200 µm). Panels (**A**–**D**) show SO 10% MA at increasing crystallization time points, while panels (**E**–**H**) depict CLO 10% MA at corresponding stages. Compared to salmon oil, cod liver oil promotes faster and more extensive crystal growth, forming larger spherulitic structures.

Figure 2 illustrates the effect of oil type and crystal growth over time for MA-based oleogels (10% MA). In SO (Figure 2A–D), crystal development progresses gradually. Initial nucleation (A, B) leads to spherulitic growth (C), eventually forming large radial aggregates with characteristic birefringence (D). In CLO (Figure 2E–H), however, crystal structures appear earlier and more extensively. The crystals in CLO grow larger over time, forming fan-shaped or layered spherulites. This faster and more expansive crystal growth in CLO may be influenced by its higher degree of saturation and lower polyunsaturated fatty acids content compared to SO, which reduces molecular interference and allows faster alignment of gelator molecules [15]. The oil matrix thus plays a critical role in directing nucleation and crystal growth, with CLO providing a more favourable medium for low-molecular-weight gelators like MA to crystallize efficiently. In contrast, our experimental observations indicated that the crystal morphology of RBW-based oleogels remained relatively consistent between SO and CLO). Both systems showed dense, needle-like crystallites forming an interwoven network. This suggests that RBW gelation is less sensitive to the oil’s compositional differences and can form similar networks across both oil types.

### 2.2. Thermal Properties

**Figure 3:**
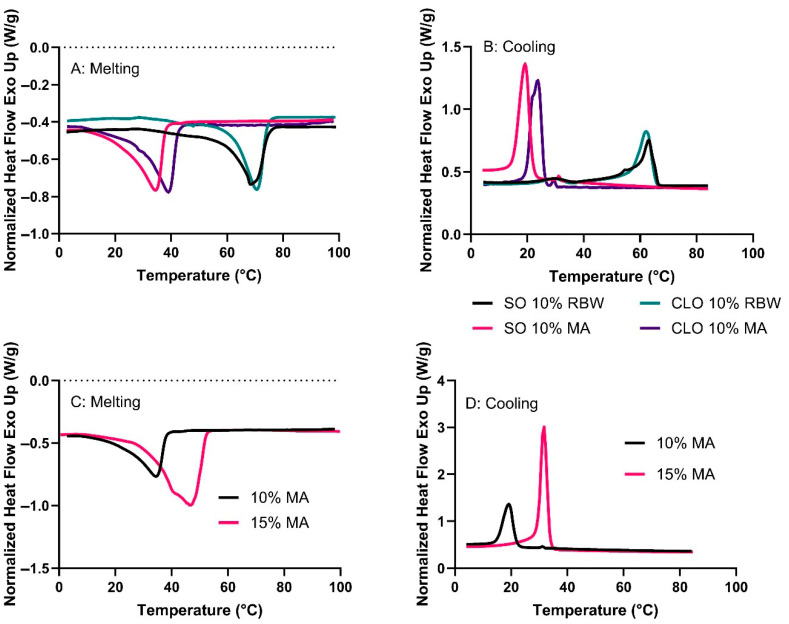
DSC thermograms of oleogels with different fish oils and gelators: (**A**,**B**) Melting and cooling behaviour of oleogels with 10% RBW or 10% MA; (**C**,**D**) Melting and cooling profiles of SO-based oleogels with 10% and 15% MA. The dotted line corresponds to zero heat flow.

Differential Scanning Calorimetry (DSC) was employed to investigate the melting and crystallization behaviours of the oleogels structured with RBW and MA in both cod liver oil and salmon oil. The melting profiles (Figure 3A,C) reveal distinct thermal characteristics depending on the gelator type and concentration. Rice bran wax-based oleogels (CLO 10% RBW and SO 10% RBW) exhibit endothermic peaks around 68–70 °C, which is consistent with the melting point and crystalline nature of RBW. These well-defined peaks reflect the presence of a structured network of densely packed wax crystals, which is a typical feature of oleogels structured by plant waxes [14,16].

Myristic acid-based oleogels of 10 wt% MA and 15 wt% MA in SO show broader melting transitions, with peak maxima around 30–35 °C and 40–45 °C, respectively (Figure 3C). This behaviour suggests that MA forms less organized, possibly polymorphic crystal structures that melt over a wider temperature range. The increase in MA concentration from 10% to 15% in SO significantly shifts the melting peak to a higher temperature, and increased enthalpic response indicates a greater crystalline mass. The thermal behaviour of these oleogels is also influenced by the oil phase. Cod liver oil-based oleogels showed slightly higher melting transitions than their SO-based oleogels. This may be attributed to SO’s higher degree of unsaturation compared with CLO, which can interfere with gelator crystallization, reducing crystal packing density and network order [17].

During cooling (Figure 3B,D), RBW-containing oleogels showed narrow, well-defined exothermic peaks in the cooling cycle at approximately 55–62 °C, depending on the oil type. These high crystallization temperatures reflect the strong nucleating ability and high melting/crystallization point of RBW, which is known to form highly ordered crystalline structures. In contrast, MA-based oleogels exhibit faster and earlier crystallization, with exothermic peaks at 25–35 °C. Increasing the MA concentration from 10% to 15% (SO system) causes a pronounced increase in peak intensity and sharpness, suggesting enhanced nucleation and growth of crystalline domains. This behaviour aligns with the lower molecular weight of MA and faster crystallization kinetics compared to waxes. However, the resulting crystals are often larger and less connected, as reported in previous reports on fatty acid-based oleogels [18]. The DSC results demonstrate that RBW forms thermally stable oleogels, which are beneficial for food products requiring structural integrity over a range of storage and processing temperatures. Myristic acid, on the other hand, allows for more rapid crystallization and earlier melting, which may be advantageous for applications where faster breakdown in the mouth or at lower temperatures is desirable.

### 2.3. Viscosity Behaviour

**Figure 4:**
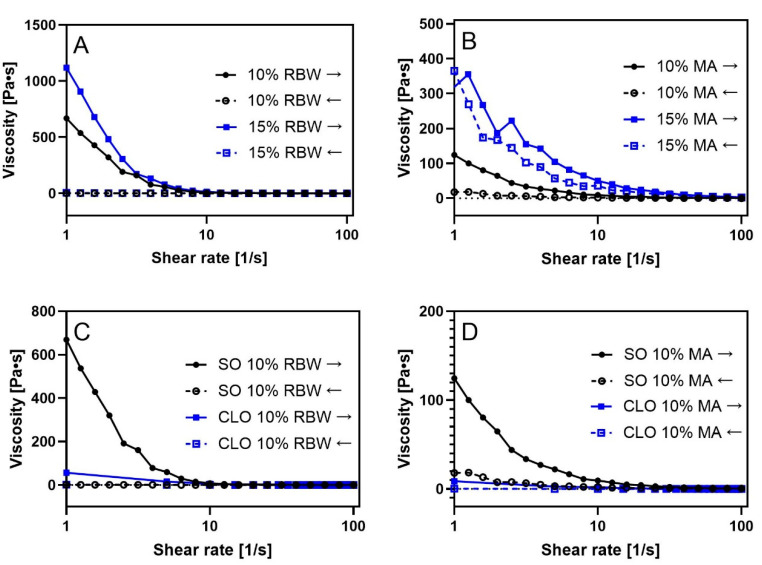
Viscosity profiles of RBW-based oleogels and MA-based oleogels in salmon oil (SO) or cod liver oil (CLO). (**A**) Viscosity vs. shear rate curves of SO-based oleogels with 10% and 15% RBW. (**B**) Viscosity vs. shear rate curves of SO-based oleogels with 10% and 15% MA. (**C**) Comparison of SO 10% RBW and CLO 10% BRW. (**D**) Comparison of SO 10% MA and CLO 10% MA. Arrows indicate the direction of shear (→ increasing; ← decreasing).

The viscosity of the oleogels structured with RBW and MA in salmon oil or cod liver oil provides important insights into their structural integrity and suitability for food applications. The viscosity–shear rate curves (Figure 4) show distinct viscosity profiles associated with gelator type, concentration, and oil medium. All oleogels exhibited shear-thinning behaviour where viscosity decreased with increasing shear rate. This is characteristic of structured systems where a three-dimensional network resists deformation under low shear but breaks down progressively under stress—a desirable feature for spreadable food products [19].

At low shear rates (1–10 1/s), the viscosity values reflect the strength of the internal network. In RBW-based oleogels (Figure 4A), a clear concentration dependence was observed. The 15% RBW oleogel showed significantly higher viscosity than 10% RBW, indicating a denser crystalline network and greater structural resistance to deformation. The difference between the increasing and decreasing shear curves suggests thixotropic behaviour, where the gel structure partially breaks down under shear and gradually recovers when shear is reduced. This property is beneficial for food applications that need to flow under force but retain structure afterward.

Myristic acid-based oleogels (Figure 4B) displayed lower overall viscosities, indicating that the network was clearly weaker than RBW-based systems. When comparing oil types (Figure 4C,D), gels in SO showed higher viscosity than those in CLO, particularly at low shear rates. This suggests that both RBW and MA form a more continuous and cohesive network in salmon oil. Interestingly, while CLO-based oleogels exhibited slightly higher melting temperatures than their SO-based oleogels, suggesting thermally stable crystalline structures (DSC data in Figure 3), their viscosity curves showed lower values. This indicates that despite the thermal robustness, the oleogel networks in CLO are less cohesive. This could be attributed to oil composition differences: CLO contains more saturated fatty acids, but also bioactives such as cholesterol and fat-soluble vitamins, which may interfere with crystal networking and reduce the structural integrity of oleogels [18].

### 2.4. Stability in Simulated Gastric and Intestinal Fluids

**Figure 5:**
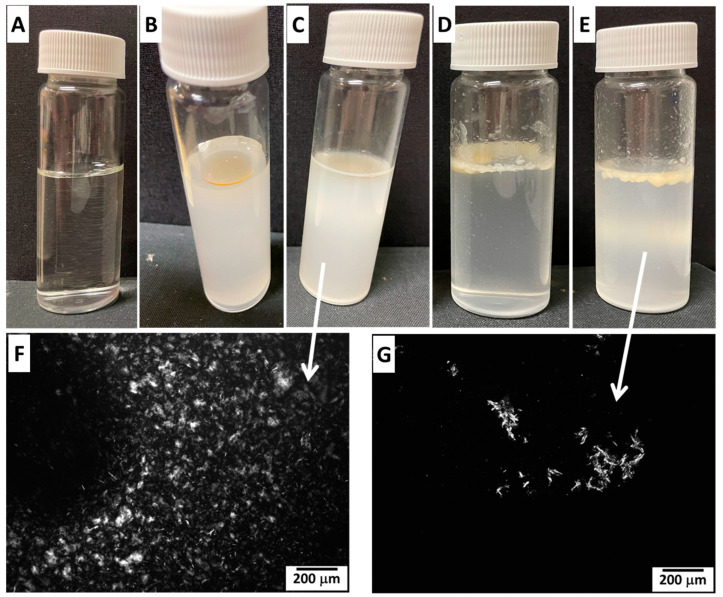
Stability of SO-based oleogels in simulated gastric fluid (SGF) and simulated intestinal fluid (SIF). (**A**) SGF/SIF control fluids; (**B**) SO 10% MA after SGF (2 h) followed by SIF (4 h); (**C**) SO 10% MA after SGF (2 h) + SIF (24 h); (**D**) SO 10% RBW after SGF (2 h) + SIF (4 h); (**E**) SO 10% RBW after SGF (2 h) + SIF (24 h). (**F**,**G**) Polarized optical microscopy (POM) images of samples (**C**,**E**), respectively, after 2 h in SGF and 24 h in SIF. All samples were gently shaken prior to microscopy to ensure homogeneity.

The stability of SO-based oleogels with 10% MA or 10% RBW was evaluated in simulated gastric fluid (SGF, 2 h) followed by exposure to simulated intestinal fluid (SIF, 4 or 24 h). Visual inspection and microscopic analysis were used to assess structural integrity and potential breakdown of the oleogel networks during digestion. When samples were left undisturbed, some phase separation was visible in the photographic images (Figure 5B–E). However, before polarized optical microscopy, all samples were gently shaken to ensure homogeneity; therefore, the aliquots analyzed by POM were representative of the overall gel system rather than a specific separated phase. After sequential exposure to SGF and SIF, RBW-based oleogels (Figure 5D,E) showed clear signs of partial disintegration. A soft, floating layer developed after both 4 and 24 h in SIF, indicating gradual breakdown of the gels. In contrast, RBW oleogels retained a more visible structure over time compared with MA-based systems. In contrast, MA oleogels (Figure 5B,C) were visibly less stable: after 24 h in SIF, no distinct gel phase remained, and the systems appeared as homogeneous dispersions, suggesting pronounced breakdown or emulsification of the oleogel network.

These visual observations were further supported by polarized optical microscopy. As shown in Figure 5F, the SO 10% MA sample (after SGF 2 h + SIF 24 h) revealed a dispersed crystalline phase, indicating that the gel network had lost its cohesive structure. Meanwhile, the corresponding RBW sample (Figure 5G) still exhibited larger and more organized crystalline domains, consistent with its relatively better physical stability. The higher stability of RBW-based oleogels in gastrointestinal conditions can be attributed to the stronger and more crystalline gel network formed by waxes, as previously demonstrated in DSC (Figure 3) and viscosity studies (Figure 4). Rice bran wax forms a robust 3D network that resists enzymatic and pH-induced degradation to a greater extent than the softer, less cohesive networks formed by MA. This behaviour is beneficial for use in applications requiring sustained lipid structuring or delivery under gastrointestinal conditions [18].

### 2.5. Oxidative Stability of Oleogels During Storage

**Figure 6:**
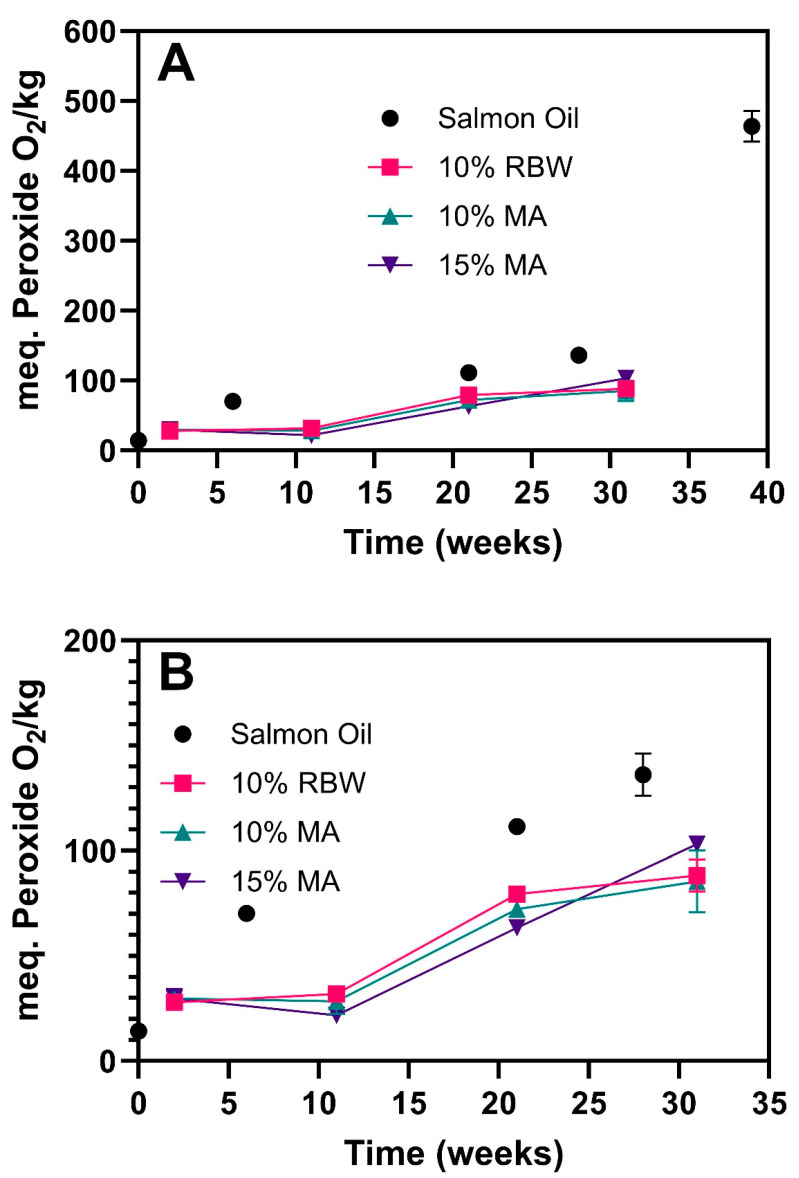
Oxidative stability of salmon oil and its oleogels during 31 weeks of storage at 5 °C. (**A**) Peroxide values (meq O_2_/kg) of salmon oil and oleogels structured with 10% RBW, 10% MA, and 15% MA; (**B**) Expanded view of the same data (0–200 meq O_2_/kg) for improved comparison between oleogels. Error bars represent standard deviations.

The oxidative stability of salmon oil and its oleogel formulations was assessed over a 31-week storage period by measuring peroxide values (PV), which serve as indicators of primary lipid oxidation. As the objective of this study was to evaluate the relative effect of oleogel composition on oxidative stability of fish oils, no additional measures were taken to prevent contact with oxygen during processing. Moreover, the samples were not flushed with an inert gas during storage, and thus all the systems studied here show an increase in oxidation over time, which is in agreement with comparable literature on fish oil oleogels [9,11]. As shown in Figure 6, all oleogel formulations, whether structured with rice bran wax or myristic acid, significantly reduced the extent of time-dependent oxidation compared to unstructured salmon oil. This is in agreement with Hwang [20] et al., who demonstrated that structuring fish oil with natural waxes (e.g., rice bran wax and soybean wax) led to significantly slower oxidation compared to unstructured oil stored at 35 °C, supporting the protective role of the crystalline network.

However, the oleogel-forming process results in oxidation compared to the base/unprocessed oil, which is also in agreement with comparable literature [9,11]. The base oil exhibited a substantial increase in peroxide value, reaching approximately 120 meq O_2_/kg by week 21, while the peroxide values of all oleogel samples remained below ~80 meq O_2_/kg throughout the storage period. These results confirm that the oleogel network helps slow down oxygen diffusion and provides a degree of protection against oxidative degradation. Interestingly, the three oleogel systems (10% RBW, 10% MA, and 15% MA) exhibited comparable oxidative stability, with no consistent differences observed among them. This suggests that both gelators, despite differing in their crystallization patterns and network morphology, are similarly effective in forming physical barriers that limit oxygen access and suppress peroxidation. This is in contrast to previously reported results, where the melting point of the wax was found to be proportional to the oxidative protection [20]. The PV results further indicate that increasing MA concentration from 10% to 15% did not improve oxidative stability. This implies that 10% MA is sufficient to enhance the oxidative resistance of the oleogel, and that higher concentrations offer no additional benefit in this regard. Instead, the increased MA content contributed more meaningfully to other functional properties such as thermal resistance and flow behaviour, as previously discussed in the DSC (Figure 3) and viscosity analyses (Figure 4), rather than enhancing oxidative protection.

## 3. Conclusions

This study demonstrated the potential of using rice bran wax and myristic acid as structuring agents to develop oleogels based on salmon oil and cod liver oil for food or nutraceutical applications. Both gelators successfully formed self-standing oleogels with distinct microstructures, thermal behaviours, and viscosity properties, influenced by gelator type, concentration, and the oil matrix. Polarized microscopy revealed that RBW formed denser and more interconnected crystal networks, while MA produced larger, less cohesive structures. These morphological differences were supported by DSC data: RBW-based oleogels exhibited higher crystallization temperatures, indicating more thermally stable crystalline networks. Viscosity measurements further confirmed that RBW structured gels had higher viscosity and stronger shear-thinning behaviour, suggesting better structural integrity compared to MA-based gels. Time-resolved microscopy further revealed that MA crystallized more rapidly and extensively in cod liver oil than in salmon oil, highlighting the sensitivity of MA to the oil matrix. In contrast, RBW-based oleogels showed relatively consistent crystal morphology in both oils, indicating that RBW forms robust and stable networks regardless of oil composition. Rice bran wax-based systems showed more resistance to structural breakdown than MA oleogels over 24 h of exposure during in vitro digestion in simulated gastric and intestinal fluids. Importantly, long-term oxidative stability studies revealed that all oleogels significantly reduced lipid peroxidation compared to unstructured salmon oil. No substantial differences were observed between RBW and MA systems in peroxide value during storage, indicating that both gelators are effective in providing oxidative protection. Increasing the MA concentration from 10% to 15% improved the oleogel’s thermal and viscosity behaviour but did not further enhance oxidative stability.

In summary, these findings highlight that both RBW and MA can serve as effective gelators for structuring marine oils, with RBW offering greater thermal and mechanical stability and MA providing a simpler alternative with acceptable performance. These oleogels offer promising potential as delivery systems for functional lipids, particularly in formulations where oxidative protection is critical.

## 4. Materials and Methods

### 4.1. Materials

Rice bran wax was supplied by Aromantic Limited Elgin, United Kingdom). Rice bran wax properties were as follows: melting point: 75–82 °C; acid value: 1–12 mg KOH g^−1^; iodine value: 5–13 g I2/100 g; saponification value: 75–90.0 mg KOH g^−1^, and free fatty acids: <10%. Myristic acid was supplied by Sigma Aldrich (St. Louis, MO, USA). Cod liver oil (CLO) (commercial fish oil—Møllers Tran Natural, Norway) was bought at supermarkets, retail pharmacies, and health food stores (Trondheim, Norway). The fish oil made from the liver of fresh 100% Arctic cod oil contains Vitamin D, Vitamin A, Vitamin E, and Omega-3 fatty acids (DHA, EPA). Salmon oil was obtained from SINTEF Ocean (Trondheim, Norway) as a gift, without an antioxidant, and was used without modification. Chloroform and methanol used for the extraction of fish oil were obtained from Sigma-Aldrich. For peroxide value (PV) analysis, the following reagents were acquired from Merck (Millipore Sigma, Merck KGaA, Munich, Germany): 37% hydrochloric acid, iron(II) sulfate heptahydrate, ammonium thiocyanate, iron(III) chloride hexahydrate, sodium sulfite, trichloroacetic acid, 2-thiobarbituric acid, and glacial acetic acid. 1,1,3,3-Tetraethoxypropane was purchased from Sigma-Aldrich.

### 4.2. Preparation of Fish Oil Oleogels

**Figure 7:**
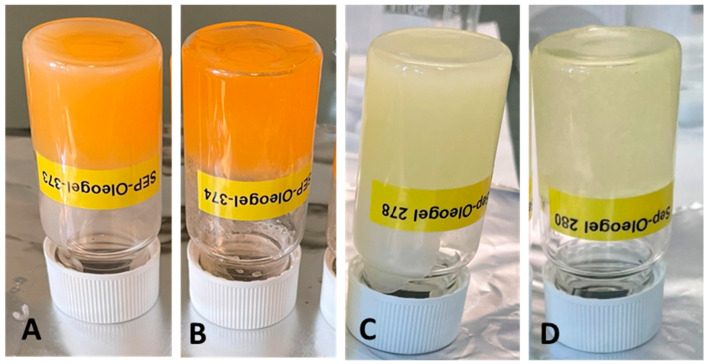
Visual appearance and structural integrity of inverted oleogels formulated with 10% rice bran wax or 10 wt% myristic acid in salmon oil or cod liver oil. (**A**) SO 10 wt% RBW, (**B**) SO 10 wt% MA, (**C**) CLO 10 wt% RBW, and (**D**) CLO 10 wt% MA. Images were taken after inverting the samples to assess gel strength and oil-binding capacity. Samples with 10 wt% gelator are presented as representative systems, since oleogels with 5 wt% gelator were not consistently self-standing, and those with 15 wt% gelator appeared visually similar to the 10 wt% formulations.

Oleogels were prepared in 10 g batches by dispersing rice bran wax (RBW) or myristic acid (MA) into salmon oil (SO) or cod liver oil (CLO) at 5, 10, or 15% (*w*/*w*). The gelators were dissolved by heating to 55 °C (MA) or 85 °C (RBW) under stirring at 300 rpm using a IKA® hotplate stirrer (IKA®-Werke GmbH & Co. KG, Staufen, Germany) for 20 min until clear solutions were obtained. Cooling the solutions to 25 °C induced gelation and yielded self-standing oleogels. Formulations were prepared in triplicate (*n* = 3), stored in glass vials, and classified as gels if they did not flow upon inversion (Figure 7). The compositions of all formulations are summarized in Table 1.

### 4.3. Microstructure Analysis

Oleogel morphology was observed using a polarized light microscope (POM) (Olympus BX43, Olympus Optical Co., Ltd., Tokyo, Japan). A drop of hot oleogel was placed on a preheated slide, covered with a preheated coverslip, and cooled to room temperature to allow crystal formation. This procedure produced thin, uniform films suitable for monitoring crystal growth. Micrographs were recorded with a digital camera attachment.

### 4.4. Thermal Behaviour Analysis

The thermal behaviour of the oleogels was investigated via differential scanning calorimetry (DSC) using a differential scanning calorimetry (DSC 2, Mettler-Toledo AG, Greifensee, Switzerland). Samples (3–5 mg) were sealed in aluminum pans (40 µL capacity) and subjected to heating and cooling scans between −20 °C and 120 °C at 10 °C/min. Melting temperature (*T_m_*) and crystallization temperature (*T_c_*) were determined using software provided with the instrument.

### 4.5. Viscosity Measurements

Viscosity measurements were performed using a Kinexus Lab+ rheometer platform 50 N NF (Nr. KNX2112, Netzsch) (NETZSCH-Gerätebau GmbH, Selb, Germany) with a CP 1/60 L1120 SS geometry (diameter 6 cm). Approximately 0.3 mL of oleogel was loaded for each measurement. The viscosity behaviour of the gels was studied by applying a shear rate sweep from 0.1 s^−1^ to 100 s^−1^, immediately followed by a reverse sweep from 100 s^−1^ back to 0.1 s^−1^. All tests were conducted at 25 °C with 10 points per decade. This protocol was applied to both SO and CLO oleogels.

### 4.6. Oxidative Stability Measurements

Oxidative stability of the fish oil oleogels was determined by measuring the peroxide values immediately after preparation of the oleogels, and after storage.

Extraction of lipids was performed according to a modified Bligh and Dyer [21] method as previously described [22,23]. Briefly, 250 mg oleogel was placed in a DT-20 blender fitted to an ULTRA-TURRAX^®^-Tube drive (IKA®-Werke GmbH & Co. KG, Staufen, Germany), and 12 mL of methanol–chloroform 2:1 (*v:v*) was added. After destruction of the oleogel in the blender, the resulting mixture was transferred to a 50 mL centrifuge tube. To obtain quantitative transfer of the oil, the blender was subsequently washed with 2 mL of DI water and 2 mL of chloroform, and the washing solution was added to the centrifuge tube. After centrifugation (10 min at 3000 RPM), the aqueous and oil phases were separated by carefully removing the aqueous (bottom) phase with a pipette, and the amount of extracted oil was weighed. A total of 3 mL of the extracted oil in chloroform was then transferred into a pre-weighed glass evaporator tube, and the chloroform was evaporated under N_2_, followed by 1 h in a vacuum oven at 25 °C. After weighing, the glass tubes were stored at 4 °C for storage stability studies and transferred to −20 °C at the designated sampling time to halt oxidation prior to further analysis of lipid oxidation. Peroxide values (PVs) for the extracts were determined by colorimetric detection of iron thiocyanate at 500 nm [24] using a plate reader (BioTek Synergy H1 hybrid multi-mode microplate reader, BioTek, Winooski, VT, USA) with measurements taken in triplicate.

### 4.7. Stability Assay in Simulated Digestion Models

The stability of the fish oil oleogels under simulated digestive conditions was assessed by exposing the gels to simulated gastric fluid (SGF) for 2 h, simulated intestinal fluid (SIF) for 4 h, or sequentially to SGF for 2 h followed by SIF for 24 h. The protocol was adapted from the guidelines for the “gastro-small intestine two step method” described by Xin et al. [25].

Simulated gastric fluid was prepared by dissolving 0.2% (*w*/*v*) NaCl and 0.32% (*w*/*v*) pepsin (from porcine gastric mucosa) in distilled water and adjusting the pH to 1.2 with 1 M HCl. Simulated intestinal fluid was prepared by dissolving 0.68% (*w*/*v*) KH_2_PO_4_ and 0.5% (*w*/*v*) pancreatin (from porcine pancreas) in distilled water, with the pH adjusted to 6.8 using 1 M NaOH. The intestinal phase duration was set to 4 h to approximate extended residence time in the small intestine, which is commonly used in digestion stability studies of lipid-based systems [25,26].

Oleogel samples (10% MA or 10% RBW in salmon oil, ~0.40 g) were weighed into glass vials and incubated with 4 mL SGF at 37 °C with gentle shaking for 2 h to simulate gastric digestion. Following this, the samples were transferred into fresh vials containing 20 mL SIF and incubated at 37 °C for either 4 h or 24 h to simulate intestinal digestion. No pH adjustment was made between phases to reflect gradual physiological transition conditions. At the end of each digestion phase, samples were visually examined for structural changes (gel breakdown, oil release, and phase separation). For microstructural analysis, a small portion of each digested sample was gently transferred to a microscope slide for polarized optical microscopy.

## Figures and Tables

**Table 1:** Composition of oleogels prepared using cod liver oil (CLO) or salmon oil (SO) structured with different concentrations of rice bran wax (RBW) or myristic acid (MA). The formulations were used for characterization of microstructure, thermal, viscosity, oxidative, and digestive properties. Detailed DSC, viscosity, and oxidative stability analyses were focused on the 10% and 15% formulations, which consistently produced stable gels. The 10% samples were selected as baseline systems, while the 15% samples were included to evaluate the effect of increasing gelator content.

Batch	Composition (%*w*/*w*)			
	Rice Bran Wax	Myristic Acid	SO	CLO
SO 5% RBW	5.00	-	95.00	-
SO 10% RBW	10.00	-	90.00	-
SO 15% RBW	15.00	-	85.00	-
SO 5% MA	-	5.00	95.00	-
SO 10% MA	-	10.00	90.00	-
SO 15% MA	-	15.00	85.00	-
CLO 10% RBW	10.00	-	-	90.00
CLO 15% RBW	15.00	-	-	85.00
CLO 10% MA	-	10.00	-	90.00
CLO 15% MA	-	15.00	-	85.00

## Data Availability

The data presented in this study are available on request from the corresponding author.
